# $H^1$-conforming finite element cochain complexes and commuting quasi-interpolation operators on Cartesian meshes

**DOI:** 10.1007/s10092-021-00409-6

**Published:** 2021-04-08

**Authors:** Francesca Bonizzoni, Guido Kanschat

**Affiliations:** 1 Department of Mathematics, University of Augsburg, Universitätsstraße 14, 86159 Augsburg, Germany; 2 Interdisciplinary Center for Scientific Computing (IWR), Heidelberg University, Klaus-Tschira-Platz 1, 69120 Heidelberg, Germany

**Keywords:** Finite element exterior calculus, de Rham complex, Commuting diagram property, Quasi-interpolation, Tensor product, 65N30

## Abstract

A finite element cochain complex on Cartesian meshes of any dimension based on the $H^1$-inner product is introduced. It yields $H^1$-conforming finite element spaces with exterior derivatives in $H^1$. We use a tensor product construction to obtain $L^2$-stable projectors into these spaces which commute with the exterior derivative. The finite element complex is generalized to a family of arbitrary order.

## Introduction

We present a family of finite element cochain complexes in $H^1({\varOmega })$ on Cartesian meshes. By adhering to a strict tensor product construction, we obtain commuting interpolation operators which are bounded on $L^2({\varOmega })$.

It has been pointed out for instance in [27] that the reliable computation of high Reynolds number incompressible flow hinges on pressure robustness of the discretization, which in turn is guaranteed by exact implementation of the divergence condition. We have demonstrated the importance of the cochain property for error estimates and adaptive mesh refinement in [19, 29]. These works have in common that they rely on divergence-conforming discontinuous Galerkin methods, which started with [12, 18]. Thus, they are consistent with the Laplacian, but not conforming in $H^1({\varOmega })$.

Due to the importance of the divergence constraint, considerable effort was put into the development of $H^1$-conforming methods with exact divergence constraint in recent years. In particular on simplicial meshes, there is a wide variety of methods. We refer the reader to the recent review [20] and the literature cited therein.

The use of the Raviart-Thomas polynomial space ${\mathbb {Q}}_{3,2}\times {\mathbb {Q}}_{2,3}$ with node functionals yielding $H^1$-conforming finite elements on rectangular meshes goes back to [5]. They already use a tensor product of Hermitian and Lagrangian interpolation on each rectangle, such that the divergence is in the space of continuous functions and cellwise in ${\mathbb {Q}}_{2,2}$.

The same polynomial space for the velocity, but with different degrees of freedom is used in [30], but with an implicitly defined pressure space. The author obtains a solution by a procedure which does not require setting up a basis for the pressure space, and thus the construction is valid. Nevertheless, the discretization spaces are bound to a specific solution scheme for the discrete problem. This was overcome later in [21] by using partly Hermitian interpolation, thus obtaining a local characterization of the pressure space. They use Hermitian degrees of freedom in vertices, but only Lagrangian on edges, such that the pressure space can be discontinuous. As a result, the velocity space does not result from tensorization of one-dimensional elements, which is one of our construction principles. Also the inf-sup stable Stokes pair of finite elements yielding diverge-free solutions in [22] does not have tensor product structure.

Discretization spaces which are $H^1$-conforming and yield diverge-free solutions have been object of the Isogeometric Analysis (IGA) literature, too. In this framework, tensor product meshes and spline-based approximation spaces are considered. We refer to [8] for IGA techniques applied to the Stokes problem, and to [14, 15] for applications to the steady and unsteady Navier-Stokes equations.

Quasi-interpolation operators for the element from [5] which commute with the divergence were first introduced in [29]. Here, we systematically reconstruct the canonical interpolation operators used there and generalize them to any space dimension and forms of any index.

While these publications were concerned with $H^1$-conforming elements with controllable divergence in two and three dimensions (reference [21] generalizes to any dimension), this paper is concerned with the full finite element cochain complex on Cartesian meshes of arbitrary dimension, such that for each finite element form $u_h\in V^k \subset H^1{\varLambda }^k({\varOmega })$ its exterior derivative is $d u_h \in V^{k+1} \subset H^1{\varLambda }^{k+1}({\varOmega })$. Based on a general lemma on the cochain property of interpolation operators, we provide commuting interpolation operators for differentiable functions as well as commuting quasi-interpolation operators which are continuous on $L^2$. For the latter, we follow the route laid out in [24–26] in one dimension and tensorize afterwards.

This article is laid out as follows: after some preliminaries in Sect. 2 we present a general construction principle for commuting interpolation operators in Sect. 3. The one-dimensional finite element cochain complex based on cubic polynomials with Hermitian interpolation is outlined in Sect. 4 and its quasi-interpolation operators are introduced in Sect. 5. The tensorization for higher-dimensional complexes is presented in Sects. 6 and 7, respectively. Sect. 8 presents the extension to higher order polynomial spaces.

## Notation and preliminaries

Following [4], we introduce the notation and definitions concerning the finite element exterior calculus that we will need throughout the paper. Let $n\ge 1$ and $0\le k\le n$ integers. We denote with ${\text {Alt}}^k{\mathbb {R}}^n$ the space of alternating *k*-linear forms on ${\mathbb {R}}^n$, with inner product $\left( \cdot ,\cdot \right) _{{\text {Alt}}^k{\mathbb {R}}^n}$. Let ${\varOmega }$ be a *n*-dimensional open bounded subset of ${\mathbb {R}}^n$. A differential *k*-form on ${\varOmega }$ is a map *u* which associates to each $x\in {\varOmega }$ an element $u\in {\text {Alt}}^k{\mathbb {R}}^n$. It can be expressed uniquely as

1 $$\begin{aligned} u=\sum _{\sigma \in {\varSigma }(k,n)} u_\sigma \, dx^\sigma , \end{aligned}$$

where $u_\sigma$ are coefficient functions defined on ${\varOmega }$, and ${\varSigma }(k,n)$ is the set of increasing maps $\{1,\ldots ,k\}\rightarrow \{1,\ldots ,n\}$. The set $\{dx^1,\ldots ,dx^n\}$ denotes the basis of ${\text {Alt}}^1{\mathbb {R}}^n=({\mathbb {R}}^n)^*$ dual to the canonical basis, and $dx^\sigma =dx^{\sigma _1}\wedge \cdots \wedge dx^{\sigma _k}\in {\text {Alt}}^k{\mathbb {R}}^n$. We denote by ${\varLambda }^k({\varOmega })$ the space of smooth differential *k*-forms, i.e., the space of *k*-forms with smooth coefficient functions.

Let $d^k:{\varLambda }^k({\varOmega })\rightarrow {\varLambda }^{k+1}({\varOmega })$ be the exterior derivative, i.e, the linear map which associates $u\in {\varLambda }^k({\varOmega })$ as in (1) to $d^k u\in {\varLambda }^{k+1}({\varOmega })$ given by

$$\begin{aligned} d^k u = \sum _{\sigma \in {\varSigma }(k,n)}\sum _{j=1}^n \frac{\partial u_\sigma }{\partial x_j}\ dx^j\wedge dx^\sigma . \end{aligned}$$

In the following, when no confusion occurs, we will denote the exterior derivative simply as *d*, suppressing the superscript *k*.

Given ${\mathscr {F}}({\varOmega })$ a space of functions defined on ${\varOmega }$, we denote with ${\mathscr {F}}{\varLambda }^k({\varOmega })$ the space of differential *k*-forms with coefficients in ${\mathscr {F}}({\varOmega })$. As examples, we mention the space of $C^m$-regular differential *k*-forms $C^m{\varLambda }^k({\varOmega })$, the space of $L^2({\varOmega })$-integrable *k*-forms $L^2{\varLambda }^k({\varOmega })$, and the space of polynomial differential *k*-forms ${\mathbb {P}}_m{\varLambda }^k({\varOmega })$.

The space $L^2{\varLambda }^k({\varOmega })$ is a Hilbert space, with inner product

$$\begin{aligned} \left( \cdot ,\cdot \right) _{L^2{\varLambda }^k}&:L^2{\varLambda }^k({\varOmega })\times L^2{\varLambda }^k({\varOmega })\rightarrow {\mathbb {R}}\nonumber \\ \left( u,v\right) _{L^2{\varLambda }^k}&=\sum _{\sigma \in {\varSigma }(k,n)}\left( u_\sigma ,v_\sigma \right) _{L^2({\varOmega })}. \end{aligned}$$

We define the space $H\!{\varLambda }^k({\varOmega })$ as

$$\begin{aligned} H\!{\varLambda }^k({\varOmega }) :=\left\{ u\in L^2{\varLambda }^k({\varOmega })\, |\, du\in L^2{\varLambda }^{k+1}({\varOmega }) \right\} . \end{aligned}$$

It is a Hilbert space, with the inner product

2 $$\begin{aligned} \left( \cdot ,\cdot \right) _{H\!{\varLambda }^k}&:H\!{\varLambda }^k({\varOmega })\times H\!{\varLambda }^k({\varOmega })\rightarrow {\mathbb {R}}\nonumber \\ \left( u,v\right) _{H\!{\varLambda }^k}&=\left( u,v\right) _{L^2{\varLambda }^k}+\left( du,dv\right) _{L^2{\varLambda }^{k+1}}. \end{aligned}$$

The extended de Rham complex is the following sequence of spaces and maps:

3 $$\begin{aligned} 0\xrightarrow {\;\;\subset \;\;}{\mathbb {R}}\xrightarrow {\;\;\subset \;\;} {\varLambda }^0({\varOmega }) \xrightarrow {\;\; d \;\;} {\varLambda }^1({\varOmega })\xrightarrow {\;\;d\;\;}\cdots \xrightarrow {\;\; d\;\;} {\varLambda }^n({\varOmega })\xrightarrow {\;\; d\;\;} 0. \end{aligned}$$

From the relation $d\circ d=0$, it follows that

$$\begin{aligned} {\text {Im}}\left( d^{k-1}\right) \subset {\text {Ker}}\left( d^{k}\right) , \end{aligned}$$

where ${\text {Im}}$ and ${\text {Ker}}$ denote the range and the kernel, respectively. In the case of a contractible domain, the sequence (3) is exact, meaning that

$$\begin{aligned} {\text {Im}}\left( d^{k-1}\right) = {\text {Ker}}\left( d^{k}\right) . \end{aligned}$$

In the case of a noncontractible domain, the codimension of ${\text {Im}}(d^{k-1})$ in ${\text {Ker}}(d^{k})$ is equal to the corresponding Betti number.

The complex

4 $$\begin{aligned} 0\xrightarrow {\;\;\subset \;\;}{\mathbb {R}}\xrightarrow {\;\;\subset \;\;} H\!{\varLambda }^0({\varOmega }) \xrightarrow {\;\; d\;\;} H\!{\varLambda }^1({\varOmega }) \xrightarrow {\;\;d\;\;}\cdots \xrightarrow {\;\; d\;\;} H\!{\varLambda }^n({\varOmega }) \xrightarrow {\;\;d\;\;}0 \end{aligned}$$

is the bounded $L^2$ de Rham complex on ${\varOmega }$.

In the same way, given $m\ge n$ integer, it is possible to construct a cochain complex with the set of spaces $\{C^{m-k}{\varLambda }^k({\varOmega }),\ k=0,\ldots ,n\}$, as follows

5 $$\begin{aligned} 0\xrightarrow {\;\;\subset \;\;} {\mathbb {R}}\xrightarrow {\;\;\subset \;\;} C^m{\varLambda }^0({\varOmega }) \xrightarrow {\;\; d\;\;} C^{m-1}{\varLambda }^1({\varOmega }) \xrightarrow {\;\;d\;\;}\cdots C^{m-n}{\varLambda }^n({\varOmega })\xrightarrow {\;\; d\;\;} 0. \end{aligned}$$

## Construction of a commuting interpolation operator

Let $0\le k<n$ be fixed, and let ${\mathbb {P}}{\varLambda }^k({\varOmega })$ and ${\mathbb {P}}{\varLambda }^{k+1}({\varOmega })$ be polynomial forms such that $d{\mathbb {P}}{\varLambda }^k({\varOmega }) \subset {\mathbb {P}}{\varLambda }^{k+1}({\varOmega })$. Denote with *r* the dimension of the range of *d*, that is, by the rank-nullity theorem, $r = \dim {\mathbb {P}}{\varLambda }^k-\dim {\text {Ker}}d$. Moreover, let $I_k:{\varLambda }^k({\varOmega })\rightarrow {\mathbb {P}}{\varLambda }^k({\varOmega })$ and $I_{k+1}:{\varLambda }^{k+1}({\varOmega })\rightarrow {\mathbb {P}}{\varLambda }^{k+1}({\varOmega })$ denote the interpolation operators defined by

6 $$\begin{aligned} I_j u = \sum _{i=1}^{\dim {\mathbb {P}}{\varLambda }^j} {\mathscr {N}}^j_i(u)\varphi ^j_i,\qquad j=k,k+1, \end{aligned}$$

where

- $\{\varphi ^j_i\}_{i=1,\ldots ,\dim {\mathbb {P}}{\varLambda }^j({\varOmega })}$ is a basis for ${\mathbb {P}}{\varLambda }^j({\varOmega })$;
- $\{{\mathscr {N}}^j_i\}_{i=1,\ldots ,\dim {\mathbb {P}}{\varLambda }^j({\varOmega })}$ is a set of node functionals in $({\varLambda }^j({\varOmega }))^*$;
- both sets are chosen such that there holds the interpolation condition
  7 $$\begin{aligned} {\mathscr {N}}^j_i(\varphi ^j_m) = \delta _{im}. \end{aligned}$$

The following lemma gives sufficient conditions on $\{\varphi ^j_i\}_i$ and $\{{\mathscr {N}}^j_i\}_i$ such that 
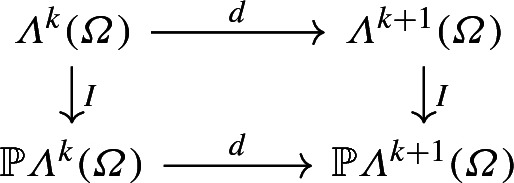
 is a commuting diagram. Here we used the convention that $I=I_k$ if its domain is ${\varLambda }^k$. It simplifies the construction of commuting quasi-interpolation operators, since it later on only requires the transformation of node functionals to perturbed cells, not the transformation of basis functions.

### Lemma 1

*With the notations introduced above, assume that the bases* $\{\varphi ^k_i\}$ *and* $\{\varphi ^{k+1}_i\}$ *fulfill*

8 $$\begin{aligned} \begin{aligned} d\varphi ^k_i&= \varphi ^{k+1}_i&\qquad i&=1,\dots ,r,\\ d\varphi ^k_i&= 0&i&=r+1,\dots ,\dim {\mathbb {P}}{\varLambda }^{k}({\varOmega }). \end{aligned} \end{aligned}$$

*Moreover, assume that the node functionals* $\{{\mathscr {N}}^k_i\}$ *and* $\{{\mathscr {N}}^{k+1}_i\}$ *fulfill for any* $u\in {\varLambda }^k({\varOmega })$

9 $$\begin{aligned} \begin{aligned} {\mathscr {N}}^{k+1}_i(d u)&= {\mathscr {N}}^k_i(u)&\qquad i&=1,\dots ,r,\\ {\mathscr {N}}^{k+1}_i(d u)&= 0&i&=r+1,\dots ,\dim {\mathbb {P}}{\varLambda }^{k+1}. \end{aligned} \end{aligned}$$

*Then, the interpolation operator* $I$ *defined in* (6) *commutes with the exterior derivative* *d*, *namely, there holds:*

$$\begin{aligned} d^k I_k u = I_{k+1} d^k u \qquad \forall u \in {\varLambda }^k({\varOmega }). \end{aligned}$$

### Proof

By linearity, we have

$$\begin{aligned} d^kI_k u = d^k\left( \sum _{i=1}^{\dim {\mathbb {P}}{\varLambda }^k}{\mathscr {N}}^k_i(u) \varphi ^k_i\right) = \sum _{i=1}^{\dim {\mathbb {P}}{\varLambda }^k}{\mathscr {N}}^k_i(u) d^k \varphi ^k_i = \sum _{i=1}^{r}{\mathscr {N}}^k_i(u) \varphi ^{k+1}_i. \end{aligned}$$

On the other hand,

$$\begin{aligned} I_{k+1} d^k u = \sum _{i=1}^{\dim {\mathbb {P}}{\varLambda }^{k+1}}{\mathscr {N}}^{k+1}_i(d^k u) \varphi ^{k+1}_i = \sum _{i=1}^{r}{\mathscr {N}}^{k+1}_i(d^ku) \varphi ^{k+1}_i. \end{aligned}$$

Employing (9) concludes the proof. $\square$

The lemma states that we can construct commuting interpolation operators in five steps:

1. Choose node functionals for ${\text {Im}}d \subset {\mathbb {P}}{\varLambda }^{k+1}$.
2. Choose node functionals for ${\mathbb {P}}{\varLambda }^k$ according to (9).
3. Choose a basis for ${\text {Im}}d \subset {\mathbb {P}}{\varLambda }^{k+1}$ such that interpolation condition (7) holds.
4. Choose a basis for ${\mathbb {P}}{\varLambda }^k$ according to (7) and (8).
5. Choose the remaining basis functions and node functionals such that (8) and (9) hold.

### Remark 1

Lemma 1 applies naturally to

$$\begin{aligned} \begin{aligned} I_k&:&C^{m-k}{\varLambda }^k({\varOmega })&\rightarrow {\mathbb {P}}{\varLambda }^k({\varOmega })\\ I_{k+1}&:&C^{m-k-1}{\varLambda }^{k+1}({\varOmega })&\rightarrow {\mathbb {P}}{\varLambda }^{k+1}({\varOmega }) \end{aligned} \end{aligned}$$

for all $m\ge k+1$, entailing the commutativity of the following diagram 
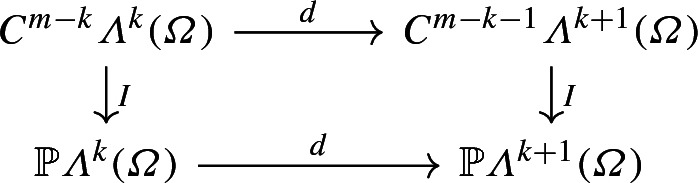


## The one-dimensional complex on the reference interval

Within this section we take ${\varOmega }$ equal to the unit interval ${\mathscr {I}}=[0,1]$, and $m=1$. Then the cochain complex (5) becomes the exact sequence

$$\begin{aligned} 0 \xrightarrow {\;\;\subset \;\;}{\mathbb {R}}\xrightarrow {\;\; \subset \;\;} C^1{\varLambda }^0({\mathscr {I}}) \xrightarrow {\;\; d \;\;} C^0{\varLambda }^1({\mathscr {I}}) \xrightarrow {\;\; d \;\;} 0. \end{aligned}$$

We discretize the spaces $C^1{\varLambda }^0({\mathscr {I}})$ and $C^0{\varLambda }^1({\mathscr {I}})$ by polynomial spaces

$$\begin{aligned} {\mathbb {P}}_3{\varLambda }^0({\mathscr {I}}) = {\mathbb {P}}_3({\mathscr {I}}) \qquad \text {and}\qquad {\mathbb {P}}_2{\varLambda }^1({\mathscr {I}}) = {\mathbb {P}}_2({\mathscr {I}}), \end{aligned}$$

respectively. In particular, we consider

- the space ${\mathbb {P}}^3{\varLambda }^0({\mathscr {I}})={\mathbb {P}}_3({\mathscr {I}})$ with the interpolation operator $I_0:C^1{\varLambda }^0({\mathscr {I}})\rightarrow {\mathbb {P}}_3{\varLambda }^0({\mathscr {I}})$ defined by the conditions ${\mathscr {N}}^0_i I_0 u = {\mathscr {N}}^0_i u$, for $i=1,2,3,4$, where the node functionals ${\mathscr {N}}^0_i:C^1{\varLambda }^0({\mathscr {I}})\rightarrow {\mathbb {R}}$ are given by a modified Hermitian interpolation:
  10 $$\begin{aligned} \begin{aligned} {\mathscr {N}}^0_1 (u)&= u'(0) \qquad&{\mathscr {N}}^0_3 (u)&= u(1)-u(0)\\ {\mathscr {N}}^0_2 (u)&= u'(1)&{\mathscr {N}}^0_4 (u)&= u(1)+u(0) \end{aligned} \end{aligned}$$
- the space ${\mathbb {P}}_2{\varLambda }^1({\mathscr {I}}) = {\mathbb {P}}_2({\mathscr {I}})$ with the interpolation operator $I_1:C^0{\varLambda }^1({\mathscr {I}})\rightarrow {\mathbb {P}}_2{\varLambda }^1({\mathscr {I}})$ defined by the conditions ${\mathscr {N}}^1_j I_1 v = {\mathscr {N}}^1_j v$, for $j=1,2,3$, where the node functionals ${\mathscr {N}}^1_j:C^0{\varLambda }^1({\mathscr {I}})\rightarrow {\mathbb {R}}$ are given by:
  11 $$\begin{aligned} \begin{aligned} {\mathscr {N}}^1_1 (v)&= v(0)\\ {\mathscr {N}}^1_2 (v)&= v(1) \end{aligned} \qquad {\mathscr {N}}^1_3 (v) = \int _{{\mathscr {I}}} v(x)\,dx. \end{aligned}$$

The second set of node functionals is a well-known alternative to Lagrange interpolation and it yields a unisolvent finite element. The basis of ${\mathbb {P}}_2({\mathscr {I}})$ dual to the set of node functionals in (11) is:

12 $$\begin{aligned} \varphi ^1_1(x)=1-4x+3x^2,\quad \varphi ^1_2(x)=-2x+3x^2,\quad \varphi ^1_3(x)=6x-6x^2. \end{aligned}$$

By straight forward computation, we obtain the following result.

### Lemma 2

*If in* ${\mathbb {P}}_3({\mathscr {I}})$ *we choose the basis*

13 $$\begin{aligned} \begin{aligned} \varphi ^0_1(x)&= x-2x^2+x^3 \qquad&\varphi ^0_3(x)&= -\tfrac{1}{2}+3x^2-2x^3\\ \varphi ^0_2(x)&= -x^2+x^3&\varphi ^0_4(x)&= \tfrac{1}{2} \end{aligned} \end{aligned}$$

*there holds* ${\mathscr {N}}^0_i(\varphi ^0_j) = \delta _{ij}$, *and the element is unisolvent.*

In this framework, the interpolation operators defined in (6) become:

$$\begin{aligned} I_0 u(x) = \sum _{i=1}^4 {\mathscr {N}}^0_i(u) \,\varphi ^0_i(x), \qquad I_1 v(x) = \sum _{j=1}^3 {\mathscr {N}}^1_j (v) \,\varphi ^1_j(x). \end{aligned}$$

The following lemma shows that $I_0$ and $I_1$ commute with the exterior derivative.

### Lemma 3

*The following diagram commutes:*
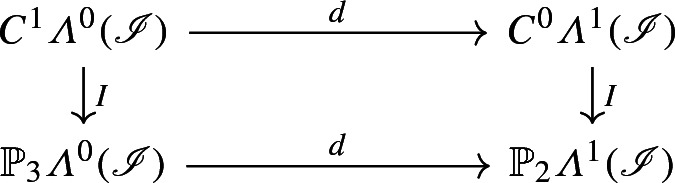
*that is, for every* $u\in C^1{\varLambda }^0({\mathscr {I}})$, *there holds*

$$\begin{aligned} d^0 I_0 u = I_1 d^0 u. \end{aligned}$$

### Proof

To prove the result, it is enough to verify that the assumptions of Lemma 1 are fulfilled. Comparing the bases in (13) and (12), we see by straightforward computation that (8) holds. In particular, $d\varphi ^0_4 \equiv 0$, such that $\varphi ^0_4$ spans the kernel of *d*. Moreover,

$$\begin{aligned} {\mathscr {N}}^1_1(du) = du(0) = u'(0) = {\mathscr {N}}^0_1(u). \end{aligned}$$

The same argument yields for ${\mathscr {N}}^1_2(du) = {\mathscr {N}}^0_2(u)$. Furthermore,

$$\begin{aligned} {\mathscr {N}}^1_3(du) = \int _{{\mathscr {I}}} du \,dx= \int _{{\mathscr {I}}} u' \,dx= u(1) - u(0) = {\mathscr {N}}^0_3(u). \end{aligned}$$

$$\square $$

### Remark 2

The set of node functionals $\{{\mathscr {N}}^0_j\}_{j=1}^4$ doesn’t contain the evaluation functionals in the end points of the interval ${\mathscr {I}}$ individually. Hence it is not immediately obvious that the interpolation operator $I_0$ generates continuity.

Nevertheless, when interpolating a function $u\in C({\mathscr {I}})$, we have

$$\begin{aligned} \begin{array}{rlrrl} I_0 u(0) &{}= {\mathscr {N}}_3(u)\varphi ^0_3(0) + {\mathscr {N}}_4(u)\varphi ^0_4(0) &{}\,=&{} -\tfrac{1}{2} \bigl [u(1)-u(0)\bigr ]+\tfrac{1}{2} \bigl [u(1)+u(0)\bigr ] &{}= u(0),\\ I_0 u(1) &{}= {\mathscr {N}}_3(u)\varphi ^0_3(1) + {\mathscr {N}}_4(u)\varphi ^0_4(1) &{}=&{} \tfrac{1}{2} \bigl [u(1)-u(0)\bigr ]+\tfrac{1}{2} \bigl [u(1)+u(0)\bigr ] &{}= u(1). \end{array} \end{aligned}$$

Thus, $I_0$ interpolates the function values at the interval end points like standard Hermite interpolation does. In particular, if several intervals are put adjacent to each other, forming a one-dimensional mesh, global $C^1$-continuity of the interpolated function is guaranteed by the standard procedure of identifying the degrees of freedom on shared vertices.

To conclude this remark: from the implementation point of view, the original node functionals for Hermite interpolation are preferred. The node functionals introduced in (10) are purely for the purpose of analysis.

### Remark 3

While we follow the finite element approach of studying cellwise properties together with degrees of freedom such as to generate the necessary global continuity, we could have adopted the global view of splines instead. There, the construction starts out with a string of intervals in one dimension and defines piecewise polynomials for instance by the technique of B-splines employed in isogeometric analysis. A tensor product cochain complex of this type was developed in [9]. Discounting the different choice of node functionals and basis functions, our complex can be viewed as a special case of theirs in one dimension and on tensor product domains. Nevertheless, as soon as multipatch domains are needed for more complex geometries in higher dimension, the local view of finite element degrees of freedom handles the “patch boundaries” in a natural way, while additional efforts are needed in isogeometric analysis.

## Quasi-interpolation operators

The node functionals introduced in Sect. 4 require point values of the first derivative of *u*. In this section we want to weaken this condition, by defining weighted node functionals, which yield quasi-interpolation operators on $L^2$.

### Node functionals on perturbed intervals

We begin by introducing perturbations of the reference interval ${\mathscr {I}}=[0,1]$. Let $0<\varrho \le \frac{1}{3}$ be a fixed parameter and ${\mathscr {I}}_\varrho = [-\varrho , 1+\varrho ]$. Choose $y_l,\,y_r\in {\mathscr {I}}_\varrho$ such that $y_l\in B_\varrho (0)$ and $y_r\in B_\varrho (1)$, where $B_\varrho (x)$ denotes the interval of radius $\varrho$ and center *x*. The perturbed interval ${{\tilde{{\mathscr {I}}}}}_{y_l,y_r} = [y_l, y_r]$ is defined as the image of the reference interval ${\mathscr {I}}$ via a monotone, possibly non-linear mapping

$$\begin{aligned} {\varPhi }_{y_l,y_r}: {\mathscr {I}}\rightarrow {{\tilde{{\mathscr {I}}}}}_{y_l,y_r} \end{aligned}$$

satisfying ${\varPhi }_{y_l,y_r}^{\prime }\equiv 1$ on $B_\varrho (0)$ and $B_\varrho (1)$. It turns out that the actual shape of ${\varPhi }$ is not needed in the definition of the quasi-interpolation operators. We transform the node functionals for ${\mathbb {P}}_3{\varLambda }^0$ in (10) as:

14 $$\begin{aligned} \begin{aligned} \widetilde{{\mathscr {N}}^0_1} (u)&= u'(y_l) \qquad&\widetilde{{\mathscr {N}}^0_3} (u)&= u(y_r)-u(y_l)\\ \widetilde{{\mathscr {N}}^0_2} (u)&= u'(y_r)&\widetilde{{\mathscr {N}}^0_4} (u)&= u(y_r)+u(y_l) \end{aligned} \end{aligned}$$

and those for ${\mathbb {P}}_2{\varLambda }^1$ in (11) as:

15 $$\begin{aligned} \begin{aligned} \widetilde{{\mathscr {N}}^1_1} (v)&= v(y_l)\\ \widetilde{{\mathscr {N}}^1_2} (v)&= v(y_r) \end{aligned} \qquad \widetilde{{\mathscr {N}}^0_3}(v) = \int _{{{\tilde{{\mathscr {I}}}}}_{y_l,y_r}} v({\tilde{x}}) \,d{\tilde{x}}. \end{aligned}$$

By proceeding as in the proof of Lemma 3, we obtain the following result.

#### Lemma 4

*The transformed node functionals* (14) *and* (15) *satisfy the following property:*

$$\begin{aligned} \widetilde{{\mathscr {N}}^1_i}(du)&=\widetilde{{\mathscr {N}}^0_i}(u), \quad i=1,2,3, \end{aligned}$$

*for all* $$u\in C^1{\varLambda }^0({\mathscr {I}}_\varrho )$$.

### Weighted node functionals

Let $\eta \in C^\infty ({\mathbb {R}})$ be the standard mollifier

$$\begin{aligned} \eta (x):=\left\{ \begin{array}{ll} C\, \exp \left( \frac{1}{|x|^2-1}\right) , &{} \text {if }|x|<1\\ 0, &{}\text {if }|x|>1 \end{array}\right. \end{aligned}$$

where the constant *C* normalizes the integral to 1. Then, the cut-off functions for the intervals $B_l:=B_\varrho (0)$ and $B_r:=B_\varrho (1)$ are given by

$$\begin{aligned} \eta _l(x):=\frac{1}{\varrho }\eta \left( \frac{x}{\varrho }\right) , \quad \eta _r(x):=\frac{1}{\varrho }\eta \left( \frac{x-1}{\varrho }\right) . \end{aligned}$$

Note that, due to normalization, it holds

$$\begin{aligned} \int _{{\mathbb {R}}} \eta _l(x) \,dx = \int _{{\mathbb {R}}} \eta _r(x) \,dx = 1, \qquad \left\| \eta _l\right\| _{L^2({\mathbb {R}})} = \left\| \eta _r\right\| _{L^2({\mathbb {R}})} = \frac{\left\| \eta \right\| _{L^2({\mathbb {R}})}}{\sqrt{\varrho }}. \end{aligned}$$

We introduce the weighted node functionals $\overline{{\mathscr {N}}^k_i}\in (C^{1-k}{\varLambda }^k({\mathscr {I}}_\varrho ))^*$ as follows:

16 $$\begin{aligned} \overline{{\mathscr {N}}^k_i}(u) = \iint _{B_l\, B_r} \eta _{l}(\xi _l)\eta _{r}(\xi _r) \widetilde{{\mathscr {N}}^k_i}(u) \, d\xi _r \, d\xi _l, \end{aligned}$$

for $k=0,1$ and all admissible values of *i*.

#### Remark 4

The normalization entails, for instance,

$$\begin{aligned} \overline{{\mathscr {N}}^0_1}(u) =\int _{B_l} \eta _{l}(\xi ) \widetilde{{\mathscr {N}}^0_1}(u) \, d\xi . \end{aligned}$$

Thus, the weighted node functionals $\overline{{\mathscr {N}}^0_1}(u)$, $\overline{{\mathscr {N}}^0_2}(u)$, $\overline{{\mathscr {N}}^1_1}(v)$ and $\overline{{\mathscr {N}}^1_2}(v)$ are characterized by the transformations of only a single end point. Therefore, on a one-dimensional mesh of several intervals, we can ensure that the result of the quasi-interpolation operator below is continuously differentiable, if we choose consistent averaging in each vertex shared by two intervals. This holds even for intervals of different length.

The remaining node functionals $\overline{{\mathscr {N}}^0_3}(u)$, $\overline{{\mathscr {N}}^0_4}(u)$ and $\overline{{\mathscr {N}}^1_3}(v)$ are truly double integrals on the balls around both end points.

### Quasi-interpolation operators

We define now the quasi-interpolation operators.

#### Definition 1

Let $\{\overline{{\mathscr {N}}^0_i}\}$, $\{\overline{{\mathscr {N}}^1_i}\}$ be the weighted node functionals as in (16), and $\{\varphi ^{0}_i\}$, $\{\varphi ^{1}_i\}$ be the basis functions on the reference element ${\mathscr {I}}$ as in (12) and (13). The quasi-interpolation operators on ${\mathscr {I}}_\varrho$ are defined as:

17 $$\begin{aligned} \begin{aligned} {\varPi }_0:C^1{\varLambda }^0({\mathscr {I}}_\varrho )&\rightarrow {\mathbb {P}}_3{\varLambda }^0({\mathscr {I}}) \qquad&u&\mapsto \sum _{i=1}^4 \overline{{\mathscr {N}}^0_i}(u) \,\varphi ^0_i,\\ {\varPi }_1 :C^0{\varLambda }^1({\mathscr {I}}_\varrho )&\rightarrow {\mathbb {P}}_2{\varLambda }^1({\mathscr {I}})&v&\mapsto \sum _{i=1}^3 \overline{{\mathscr {N}}^1_i}(v) \,\varphi ^1_i. \end{aligned} \end{aligned}$$

Furthermore, for later convenience, we extend both operators such that

18 $$\begin{aligned} {\varPi }_0:C^0{\varLambda }^1({\mathscr {I}}_\varrho ) \rightarrow 0, \qquad {\varPi }_1:C^1{\varLambda }^0({\mathscr {I}}_\varrho ) \rightarrow 0. \end{aligned}$$

We show now that the quasi-interpolation operators ${\varPi }_0$ and ${\varPi }_1$ are well-defined and bounded on $L^2({\mathscr {I}}_\varrho )$.

#### Theorem 1

*The quasi-interpolation operators admit the following estimates:*

$$\begin{aligned} \left\| {\varPi }_0 u\right\| _{L^2{\varLambda }^0({\mathscr {I}}_\varrho )} \le C_{{\varPi }_0} \left\| u\right\| _{L^2{\varLambda }^0({\mathscr {I}}_\varrho )}, \quad \forall u\in L^2{\varLambda }^0({\mathscr {I}}_\varrho ),\\ \left\| {\varPi }_1 v\right\| _{L^2{\varLambda }^1({\mathscr {I}}_\varrho )} \le C_{{\varPi }_1} \left\| v\right\| _{L^2{\varLambda }^1({\mathscr {I}}_\varrho )}, \quad \forall v\in L^2{\varLambda }^1({\mathscr {I}}_\varrho ), \end{aligned}$$

*where* $C_{{\varPi }_0},\ C_{{\varPi }_1}$ *are positive constants depending on* $\varrho$, *but independent of* *u* *and* *v*.

#### Proof

It is enough to show that the weighted node functionals $\overline{{\mathscr {N}}^k_i}$, for $k=0,1$, and for all admissible values of *i*, are bounded on $L^2$. First, for $u\in L^2({\varOmega })$

$$\begin{aligned} \left| \overline{{\mathscr {N}}^1_1} v\right|&= \left| \int _{B_l} \int _{B_r} \eta _l(\xi _l)\eta _r(\xi _r) v(\xi _l)\,d\xi _r\,d\xi _l\right| = \left| \int _{B_l} \eta _l(\xi _l)v(\xi _l)\,d\xi _l\right| \\&\le \left\| \eta _l\right\| _{L^2(B_l)} \left\| v\right\| _{L^2(B_l)} \le \varrho ^{-{1}/{2}}\left\| \eta \right\| _{L^2({\mathbb {R}})} \left\| v\right\| _{L^2({\mathscr {I}}_\varrho )}. \end{aligned}$$

This argument immediately transfer to $\overline{{\mathscr {N}}^1_2}$. For $\overline{{\mathscr {N}}^0_3}$ (and with appropriate modification for $\overline{{\mathscr {N}}^0_4}$) we obtain by the same means

$$\begin{aligned} \left| \overline{{\mathscr {N}}^0_3} u\right|&= \left| \int _{B_l} \int _{B_r} \eta _l(\xi _l)\eta _r(\xi _r) \bigl (u(\xi _r) - u(\xi _l)\bigr )\,d\xi _r\,d\xi _l\right| \\&= \left| \int _{B_r} \eta _r(\xi _r)u(\xi _r)\,d\xi _r - \int _{B_l} \eta _l(\xi _l)u(\xi _l)\,d\xi _l\right| \\&\le 2\, \varrho ^{-{1}/{2}}\left\| \eta \right\| _{L^2({\mathbb {R}})} \left\| u\right\| _{L^2({\mathscr {I}}_\varrho )}. \end{aligned}$$

For the integral node value we observe

$$\begin{aligned} \left| \overline{{\mathscr {N}}^1_3} v\right|&= \left| \int _{B_l} \int _{B_r} \int _{{{\tilde{{\mathscr {I}}}}}} \eta _l(\xi _l)\eta _r(\xi _r) v({\tilde{x}})\,d{\tilde{x}}\,d\xi _r\,d\xi _l\right| \\&\le \int _{B_l} \int _{B_r} \int _{{{\tilde{{\mathscr {I}}}}}} \eta _l(\xi _l)\eta _r(\xi _r) \left| v({\tilde{x}})\right| \,d{\tilde{x}}\,d\xi _r\,d\xi _l\\&\le \left\| v\right\| _{L^1({\mathscr {I}}_\varrho )} \int _{B_l} \int _{B_r} \eta _l(\xi _l)\eta _r(\xi _r)\,d\xi _r\,d\xi _l\\&\le \sqrt{1+2\varrho } \left\| v\right\| _{L^2({\mathscr {I}}_\varrho )}. \end{aligned}$$

Finally, we estimate the degrees of freedom involving derivatives using integration by parts, for instance

19 $$\begin{aligned} \begin{aligned} \left| \overline{{\mathscr {N}}^0_1} u\right|&= \left| \int _{B_l}\int _{B_r}\eta _l(\xi _l)\,\eta _r(\xi _r)u'(\xi _l)\,d\xi _r\,d\xi _l\right| = \left| \int _{B_l}\eta _l(\xi _l)u'(\xi _l)\,d\xi _l\right| \\&= \left| \int _{B_l}\eta _l'(\xi _l) u(\xi _l)\,d\xi _l\right| \le \varrho ^{-{3}/{2}}\left\| \eta '\right\| _{L^2({\mathbb {R}})} \left\| u\right\| _{L^2({\mathscr {I}}_\varrho )}. \end{aligned} \end{aligned}$$

The statement is then proved, with

$$\begin{aligned} C_{{\varPi }_0}&= \varrho ^{-3/2} \left\| \eta '\right\| _{L^2({\mathbb {R}})} \left( \left\| \varphi ^0_1\right\| _{L^2({\mathscr {I}}_\varrho )}+\left\| \varphi ^0_2\right\| _{L^2({\mathscr {I}}_\varrho )}\right) \\&\quad + 2\varrho ^{-1/2}\left\| \eta \right\| _{L^2({\mathbb {R}})} \left( \left\| \varphi ^0_3\right\| _{L^2({\mathscr {I}}_\varrho )}+\left\| \varphi ^0_4\right\| _{L^2({\mathscr {I}}_\varrho )}\right) ,\\ C_{{\varPi }_1}&= \varrho ^{-1/2}\left\| \eta \right\| _{L^2({\mathbb {R}})} \left( \left\| \varphi ^1_1\right\| _{L^2({\mathscr {I}}_\varrho )}+\left\| \varphi ^1_2\right\| _{L^2({\mathscr {I}}_\varrho )}\right) \\&\quad + \sqrt{1+2\varrho }\left\| \varphi ^1_3\right\| _{L^2({\mathscr {I}}_\varrho )}. \end{aligned}$$

$$\square $$

#### Remark 5

Showing boundedness in $L^2$ may not seem well motivated. Indeed, for many applications, boundedness in $H^1$ or $H{\varLambda }$ may be sufficient. We point out though, that the tensor product space $H^1({\mathscr {I}})\otimes \dots \otimes H^1({\mathscr {I}})$ is a proper subspace of $H^1({\mathscr {I}}\times \dots \times {\mathscr {I}})$. Thus, $H^1$-stability in one dimension does not immediately imply the same for the tensor product, while we have equality of the corresponding $L^2$-spaces by Fubini’s theorem (see Remark 6 below). Note that stability with respect to stronger norms follows by stability in $L^2$.

On the other hand, we could have considered stability in the even weaker $L^1$-norm as in [13]. We did not do so since we use readily available strong results on tensor products of Hilbert spaces. These arguments, if they hold at all, would become more complicated for a space like $L^1$.

The following lemma shows that the quasi-interpolation operators ${\varPi }_0$ and ${\varPi }_1$ are co-chain operators.

#### Lemma 5

*The exterior derivative and the quasi-interpolation operators in* (17) *commute, namely, for all* $u\in H{\varLambda }^0({\mathscr {I}}_\varrho )$ *there holds*

$$\begin{aligned} d^0 {\varPi }_0 u = {\varPi }_1 d^0 u. \end{aligned}$$

#### Proof

From Lemma 4 it follows immediately that for all $u\in H{\varLambda }^0({\mathscr {I}}_\varrho )$

$$\begin{aligned} \overline{{\mathscr {N}}^1_i}(du)&=\overline{{\mathscr {N}}^0_i}(u), \qquad i=1,2,3. \end{aligned}$$

Thus, the assumptions of Lemma 1 for ${\varPi }_0$ and ${\varPi }_1$ are fulfilled. Since the basis functions are the same as for the canonical interpolation operator *I*, the result follows. $\square$

We note that ${\varPi }_0$ and ${\varPi }_1$ are not interpolation operators in the classical sense. In particular, they do not act as the identity on their range. They share this with the classical quasi-interpolation operators in [11, 13, 28] as well as the commuting ones in [10, 25]. By using a trick from [26], we can define new operators ${{\hat{{\varPi }}}}_0$ and ${{\hat{{\varPi }}}}_1$ which have the projection property.

To this end, let us first highlight the dependence of ${\varPi }_k$ on the size of the intervals $B_\varrho$ by writing ${\varPi }_k(\varrho )$ for $k=0,1$. By the definition of the node functionals, we have for any polynomial $p\in {\mathbb {P}}{\varLambda }^k$

$$\begin{aligned} {\varPi }_k(\varrho ) p \rightarrow I_k p \qquad \text {as}\qquad \varrho \rightarrow 0. \end{aligned}$$

Since $I_k$ acts as identity on ${\mathbb {P}}{\varLambda }^k$ the operator ${\varPi }_k$ is invertible on this space for sufficiently small $\varrho$. Then, we can set

$$\begin{aligned} {{\hat{{\varPi }}}}_k = \bigl ({\varPi }_{k|{\mathbb {P}}{\varLambda }^k}\bigr )^{-1} {\varPi }_k. \end{aligned}$$

## Tensor complex in *n* dimensions

We start the section with a brief introduction to the tensor product of cochain complexes. Then, we detail two particular cases: the tensorization of the $L^2$ de Rahm complex (4) on ${\mathscr {I}}_\varrho$, and the tensorization of the finite element complex on ${\mathscr {I}}$ introduced in Sect. 4.

### Introduction to the tensor product of cochain complexes

Let $S\subset {\mathbb {R}}^n$ and $T\subset {\mathbb {R}}^m$ be two open bounded domains, and let $V\subset H{\varLambda }^k(S)$ and $W\subset H{\varLambda }^\ell (T)$ be Hilbert spaces of differential forms, with inner products $\left( \cdot ,\cdot \right) _{H{\varLambda }^k}$ and $\left( \cdot ,\cdot \right) _{H{\varLambda }^\ell }$, respectively (see (2)). Following [23, Chapter 2] we recall the definition of the tensor product $V\otimes W$.

Given two differential forms $v\in V$ and $w\in W$, with

$$\begin{aligned} v=\sum _{\sigma \in {\varSigma }(k,n)}v_\sigma dx^\sigma ,\quad w=\sum _{\tau \in {\varSigma }(\ell ,m)}w_\tau dx^\tau , \end{aligned}$$

their tensor product $v\otimes w$ is the $(k+\ell )$-form, expressed in coordinates as

20 $$\begin{aligned} v\otimes w=\sum _{\begin{array}{c} \sigma \in {\varSigma }(k,n)\\ \tau \in {\varSigma }(\ell ,m) \end{array}} v_\sigma \otimes w_\tau \, dx^\sigma \!\!\wedge dx^\tau . \end{aligned}$$

Denote with ${\mathscr {E}}$ the set of finite linear combinations of tensor product differential forms as in (20). We define an inner product on ${\mathscr {E}}$ as

21 $$\begin{aligned} \left( v_1\otimes w_1,v_2\otimes w_2\right) _{H{\varLambda }^k\otimes H{\varLambda }^\ell } =\left( v_1,v_2\right) _{H{\varLambda }^k} \left( w_1,w_2\right) _{H{\varLambda }^\ell }, \end{aligned}$$

and we extend it by linearity to ${\mathscr {E}}$. The tensor product space $V\otimes W$ is the Hilbert space obtained as completition of ${\mathscr {E}}$ under the inner product (21). If $\{\zeta _i\}$ and $\{\xi _j\}$ are orthonormal bases of the Hilbert spaces *V* and *W*, respectively, then the set $\{\zeta _i\otimes \xi _j\}$ is an othonormal basis for $V\otimes W$, which we refer to as rank-one basis since it consists of elements of tensor rank one.

Following [2], we recall the definition of tensor product of complexes of differential forms. Let there be given two complexes $S\subset {\mathbb {R}}^n$ $T\subset {\mathbb {R}}^m$

22 $$\begin{aligned} \begin{array}{ccccccccccccc} 0&{}\xrightarrow {\;\;\subset \;\;}&{}{\mathbb {R}}&{}\xrightarrow {\;\;\subset \;\;}&{} V^0 &{}\xrightarrow {\;\;d\;\;}&{}V^1 &{}\xrightarrow {\;\;d\;\;}&{}\cdots &{}\xrightarrow {\;\;d\;\;}&{} V^n &{}\xrightarrow {\;\;d\;\;}&{}0\\ 0&{}\xrightarrow {\;\;\subset \;\;}&{}{\mathbb {R}}&{}\xrightarrow {\;\;\subset \;\;}&{} W^0 &{}\xrightarrow {\;\;d\;\;}&{}W^1 &{}\xrightarrow {\;\;d\;\;}&{}\cdots &{}\xrightarrow {\;\;d\;\;}&{} W^m &{}\xrightarrow {\;\;d\;\;}&{}0 \end{array} \end{aligned}$$

which are subcomplexes of the $L^2$ de Rham complex of $S\subset {\mathbb {R}}^n$ and $T\subset {\mathbb {R}}^m$, respectively, meaning that $V^k\subset H{\varLambda }^k(S)$ and $d(V^k)\subset V^{k+1}$ for all $k=0,\ldots ,n$, and $W^j\subset H{\varLambda }^j(T)$ and $d(W^j)\subset W^{j+1}$, for all $j=0,\ldots ,m$. The tensor product of the two complexes in (22) is the complex

23 $$\begin{aligned} 0\xrightarrow {\;\;\subset \;\;}{\mathbb {R}}\xrightarrow {\;\;\subset \;\;}(V\otimes W)^0 \xrightarrow {\;\;d\;\;}(V\otimes W)^1 \xrightarrow {\;\;d\;\;}\cdots \xrightarrow {\;\;d\;\;}(V\otimes W)^{m+n} \xrightarrow {\;\;d\;\;}0,\nonumber \\ \end{aligned}$$

where the space $(V\otimes W)^k$ is defined as

24 $$\begin{aligned} (V\otimes W)^k:=\bigoplus _{i+j=k} \left( V^i\otimes W^j\right) ,\quad k=0,\ldots ,m+n, \end{aligned}$$

and the exterior derivative $d:(V\otimes W)^k\rightarrow (V\otimes W)^{k+1}$ is defined as

25 $$\begin{aligned} d^k(u\otimes v) = d^i u\otimes v + (-1)^i u \otimes d^j v, \quad u\in V^i,\ v\in W^j,\ i+j=k. \end{aligned}$$

Note that the complex (23) is a subcomplex of the de Rham complex on the Cartesian product $S\times T$. This construction generalizes to the tensor product of any finite number of subcomplexes of the $L^2$ de Rham complex.

### Tensorization of the $L^2$ de Rham complex

We detail now the particular case where $S=T={\mathscr {I}}_\varrho \subset {\mathbb {R}}$, and the complexes in (22) coincide with the $L^2$ de Rham complex on ${\mathscr {I}}_\varrho$ (see (4))

26 $$\begin{aligned} 0\xrightarrow {\;\;\subset \;\;} {\mathbb {R}}\xrightarrow {\;\;\subset \;\;} H{\varLambda }^0({\mathscr {I}}_\varrho ) \xrightarrow {\;\; d\;\;} H{\varLambda }^1({\mathscr {I}}_\varrho ) \xrightarrow {\;\; d\;\;} 0. \end{aligned}$$

The tensorization of the complex (26) with itself gives the following complex on the square ${\mathscr {I}}_\varrho \times {\mathscr {I}}_\varrho$

27 $$\begin{aligned} 0\xrightarrow {\;\;\subset \;\;}{\mathbb {R}}\xrightarrow {\;\;\subset \;\;}\left( H{\varLambda }\otimes H{\varLambda }\right) ^{0} \xrightarrow {\;\;d\;\;}\left( H{\varLambda }\otimes H{\varLambda }\right) ^{1} \xrightarrow {\;\;d\;\;}\left( H{\varLambda }\otimes H{\varLambda }\right) ^{2} \xrightarrow {\;\;d\;\;}0,\nonumber \\ \end{aligned}$$

where, in accordance with (24), we have

28 $$\begin{aligned} \left( H{\varLambda }\otimes H{\varLambda }\right) ^{k}:=\bigoplus _{\begin{array}{c} i+j=k\\ i,j=0,1 \end{array}} H{\varLambda }^i\otimes H{\varLambda }^j, \quad k=0,1,2. \end{aligned}$$

In particular, we have

$$\begin{aligned} \begin{aligned} \left( H{\varLambda }\otimes H{\varLambda }\right) ^{0}&= H{\varLambda }^0 \otimes H{\varLambda }^0,\\ \left( H{\varLambda }\otimes H{\varLambda }\right) ^{1}&= \left( H{\varLambda }^0 \otimes H{\varLambda }^1\right) \oplus \left( H{\varLambda }^1 \otimes H{\varLambda }^0\right) ,\\ \left( H{\varLambda }\otimes H{\varLambda }\right) ^{2}&= H{\varLambda }^1 \otimes H{\varLambda }^1. \end{aligned} \end{aligned}$$

The exterior derivative $d:\left( H{\varLambda }\otimes H{\varLambda }\right) ^{k}\rightarrow \left( H{\varLambda }\otimes H{\varLambda }\right) ^{k+1}$ is defined in Eq. (25). In particular, it holds

$$\begin{aligned} \begin{aligned} d^0 (u_0\otimes v_0)&= d^0 u_0\otimes v_0 + u_0\otimes d^0 v_0,\quad&\forall \, u_0\otimes v_0&\in H{\varLambda }^0\otimes H{\varLambda }^0,\\ d^1 (u_0\otimes v_1)&= d^0 u_0\otimes v,&\forall \, u_0\otimes v_1&\in H{\varLambda }^0\otimes H{\varLambda }^1,\\ d^1 (u_1\otimes v_0)&= u_1\otimes d^0 v_0,&\forall \, u_1\otimes v_0&\in H{\varLambda }^1\otimes H{\varLambda }^0. \end{aligned} \end{aligned}$$

Formula (27) and (28) generalize to the *n*-fold tensor product, leading to the following complex on the *n*-dimensional hypercube ${\mathscr {I}}_\varrho ^{\times n}$

$$\begin{aligned} 0\xrightarrow {\;\;\subset \;\;}{\mathbb {R}}\xrightarrow {\;\;d\;\;}(H{\varLambda }^{\otimes n})^{0} \xrightarrow {\;\;d\;\;}(H{\varLambda }^{\otimes n})^{1} \xrightarrow {\;\;d\;\;}\cdots \xrightarrow {\;\;d\;\;}(H{\varLambda }^{\otimes n})^{n} \xrightarrow {\;\;d\;\;}0. \end{aligned}$$

The space $(H{\varLambda }^{\otimes n})^{k}$, for $k=0,\ldots ,n$, is defined as

29 $$\begin{aligned} (H{\varLambda }^{\otimes n})^{k}:=\bigoplus _{{\mathbf {i}}\in \chi _k} H{\varLambda }^{i_1}\otimes \cdots \otimes H{\varLambda }^{i_n}. \end{aligned}$$

Here we employ an alternative representation of ${\varSigma }(k,n)$ by characteristic vectors, where the binary vector ${\mathbf {i}}$ selects *k* out of the *n* fibers, and is thus taken from the set

30 $$\begin{aligned} \chi _k:=\left\{ \varvec{\ell }=(\ell _1,\ldots ,\ell _n)\in \{0,1\}^n \;\big |\;\sum _{j=1}^n\ell _j=k\right\} . \end{aligned}$$

Note that the tensor product space $(H{\varLambda }^{\otimes n})^{k}$ is a proper, dense subspace of the space $H{\varLambda }^k({\mathscr {I}}_\varrho ^{\times n})$. For $H^1 = H{\varLambda }^0$, see [17, Section 3.4.2], for the other spaces, note that $(C^\infty {\varLambda }^{\otimes n})^{k}$ is dense in $(H{\varLambda }^{\otimes n})^{k}$ as well as in $H{\varLambda }^k({\mathscr {I}}_\varrho ^{\times n})$.

By straightforward computations, and making use of (25), we derive the following formula for the exterior derivative $d:(H{\varLambda }^{\otimes n})^{k}\rightarrow (H{\varLambda }^{\otimes n})^{k+1}$:

31 $$\begin{aligned} d^k(u_1\otimes \cdots \otimes u_n)= \sum _{j=1}^n \theta _j\, (u_1\otimes \cdots \otimes d u_j\otimes \cdots \otimes u_n), \end{aligned}$$

where $\theta _j\in \{-1,1\}$ is defined as $\theta _j:=(-1)^{\sum _{\ell =1}^{j-1} i_\ell }$.

### Tensorization of the finite element complex

We focus now on the finite element complex

32 $$\begin{aligned} 0\xrightarrow {\;\;\subset \;\;}{\mathbb {R}}\xrightarrow {\;\;\subset \;\;}{\mathbb {P}}_3{\varLambda }^0({\mathscr {I}}) \xrightarrow {\;\;d\;\;} {\mathbb {P}}_2{\varLambda }^1({\mathscr {I}}) \xrightarrow {\;\;d\;\;}0 \end{aligned}$$

with node functionals as in (16). We write the generic ${\mathbb {P}}{\varLambda }$ to refer to either space of polynomial forms, with the understanding that ${\mathbb {P}}{\varLambda }^0 \equiv {\mathbb {P}}_3{\varLambda }^0$ and ${\mathbb {P}}{\varLambda }^1 \equiv {\mathbb {P}}_2{\varLambda }^1$.

Applying the tensor product construction to (32), we find the following tensor product complex on ${\mathscr {I}}^{\times n}$

33 $$\begin{aligned} 0 \xrightarrow {\;\;\subset \;\;}{\mathbb {R}}\xrightarrow {\;\;\subset \;\;}({\mathbb {P}}{\varLambda }^{\otimes n})^{0} \xrightarrow {\;\;d\;\;} ({\mathbb {P}}{\varLambda }^{\otimes n})^{1} \xrightarrow {\; d\;}\cdots ({\mathbb {P}}{\varLambda }^{\otimes n})^{n} \xrightarrow {\;\;d\;\;} 0, \end{aligned}$$

where the space $({\mathbb {P}}{\varLambda }^{\otimes n})^{k}$, for $k=0,\ldots ,n$, is defined as

34 $$\begin{aligned} ({\mathbb {P}}{\varLambda }^{\otimes n})^{k} = \bigoplus _{{\mathbf {i}}\in \chi _k} {\mathbb {P}}{\varLambda }^{i_1}\otimes \cdots \otimes {\mathbb {P}}{\varLambda }^{i_n}, \end{aligned}$$

the characteristic vectors $\chi _k$ being introduced in (30). The exterior derivative $d:({\mathbb {P}}{\varLambda }^{\otimes n})^{k}\rightarrow ({\mathbb {P}}{\varLambda }^{\otimes n})^{k+1}$ is as in (31).

Using the bases $\{\varphi ^i_j\}$ in one dimension, the rank-one basis of the tensor product space $({\mathbb {P}}{\varLambda }^{\otimes n})^{k}$ consists of polynomial forms like

$$\begin{aligned} \varphi ^{{\mathbf {i}}}_{{\mathbf {j}}} = \varphi ^{i_1}_{j_1} \otimes \dots \varphi ^{i_n}_{j_n} \end{aligned}$$

where ${\mathbf {i}}$ is from the set $\chi _k$ of characteristic vectors of permutations and the components of ${\mathbf {j}}$ enumerate the polynomial basis. The tensor product construction yields node functionals for $({\mathbb {P}}{\varLambda }^{\otimes n})^{k}$ of the form ${\mathscr {N}}^{{\mathbf {i}}}_{{\mathbf {j}}}={\mathscr {N}}^{i_1}_{j_1}\otimes \cdots \otimes {\mathscr {N}}^{i_n}_{j_n}$ defined on a rank-one tensor $f_1\otimes \dots \otimes f_n$ of suitable forms as

$$\begin{aligned} {\mathscr {N}}^{{\mathbf {i}}}_{{\mathbf {j}}}(f_1\otimes \dots \otimes f_n) = {\mathscr {N}}^{i_1}_{j_1}(f_1)\cdots {\mathscr {N}}^{i_n}_{j_n}(f_n), \end{aligned}$$

and extended to the whole space by linearity. Note that the one-dimensional interpolation conditions immediately yield

$$\begin{aligned} {\mathscr {N}}^{{\mathbf {i}}}_{{\mathbf {j}}}(\varphi ^{{\mathbf {l}}}_{{\mathbf {m}}}) = \delta _{{\mathbf {i}},{\mathbf {l}}} \delta _{{\mathbf {j}},{\mathbf {m}}}, \end{aligned}$$

which in turn implies duality of the two bases and thus unisolvence of the tensor product element.

Thus, the complex in (33) is a complex of finite element differential forms.

## Tensor product of quasi-interpolation operators

### Introduction to the tensor product of operators on Hilbert spaces

We start recalling the definition and some properties of the tensor product of operators on Hilbert spaces (see [23]).

#### Definition 2

Let $V,\,W$ be two Hilbert spaces, and let $F:V\rightarrow V^\prime$ and $G:W\rightarrow W^\prime$ be continuous operators. The tensor product operator $F\otimes G:V\otimes W\rightarrow V^\prime \otimes W^\prime$ is defined on functions of the type $v\otimes w$ as

$$\begin{aligned} (F\otimes G)(v\otimes w)=F(v) \otimes G(w), \end{aligned}$$

and is then extended by linearity and density.

In [23, Chapter 8] the authors prove the following result.

#### Lemma 6

*Let the spaces* $V,\, W$ *and the operators* $F,\, G$ *be as in Definition* 2. *Then, the tensor product operator* $F\otimes G$ *is bounded. In particular, it holds*

$$\begin{aligned} \left\| F\otimes G\right\| _{{\mathscr {L}}(V\otimes W,V^\prime \otimes W^\prime )} =\left\| F\right\| _{{\mathscr {L}}(V,V^\prime )} \left\| G\right\| _{{\mathscr {L}}(W,W^\prime )}. \end{aligned}$$

Definition 2 and Lemma 6 generalize to the tensor product of any finite number of bounded operators on Hilbert spaces.

### Commuting quasi-interpolation operators in *n* dimensions

Let us take $F=G={\varPi }_0$, where ${\varPi }_0$ is the commuting quasi-interpolation operator in one dimension from Sect. 5.3. Using Definition 2 and the Riesz representation theorem, we define the tensor product operator

$$\begin{aligned} {\varPi }_0\otimes {\varPi }_0:L^2{\varLambda }^0\otimes L^2{\varLambda }^0 \rightarrow {\mathbb {P}}_3{\varLambda }^0\otimes {\mathbb {P}}_3{\varLambda }^0. \end{aligned}$$

In the same way, we define

$$\begin{aligned} {\varPi }_0\otimes {\varPi }_1&:L^2{\varLambda }^0\otimes L^2{\varLambda }^1 \rightarrow {\mathbb {P}}_3{\varLambda }^0\otimes {\mathbb {P}}_2{\varLambda }^1,\\ {\varPi }_1\otimes {\varPi }_0&:L^2{\varLambda }^1\otimes L^2{\varLambda }^0 \rightarrow {\mathbb {P}}_2{\varLambda }^1\otimes {\mathbb {P}}_3{\varLambda }^0,\\ {\varPi }_1\otimes {\varPi }_1&:L^2{\varLambda }^1\otimes L^2{\varLambda }^1 \rightarrow {\mathbb {P}}_2{\varLambda }^1\otimes {\mathbb {P}}_2{\varLambda }^1. \end{aligned}$$

#### Definition 3

Given the commuting quasi-interpolation operators in one dimension from Sect. 5.3, we define the tensor product quasi-interpolator in two dimensions for $k=0,1,2$, namely

$$\begin{aligned} {\varPi }^{\otimes 2}_{k}:(L^2{\varLambda }^{\otimes 2})^{k}\rightarrow ({\mathbb {P}}{\varLambda }^{\otimes 2})^{k} \end{aligned}$$

by

$$\begin{aligned} \begin{aligned} {\varPi }^{\otimes 2}_{0}&={\varPi }_0\otimes {\varPi }_0,\\ {\varPi }^{\otimes 2}_{1}&={\varPi }_0\otimes {\varPi }_1 + {\varPi }_1\otimes {\varPi }_0,\\ {\varPi }^{\otimes 2}_{2}&={\varPi }_1\otimes {\varPi }_1, \end{aligned} \end{aligned}$$

where the space $({\mathbb {P}}{\varLambda }^{\otimes 2})^{k}$ has been defined in (34), and the space $(L^2{\varLambda }^{\otimes 2})^{k}$ is defined as

$$\begin{aligned} (L^2{\varLambda }^{\otimes 2})^{k} =\bigoplus _{\begin{array}{c} i+j=k\\ i,j=0,1 \end{array}} L^2{\varLambda }^{i}\otimes L^2{\varLambda }^{j}, \quad k=0,1,2. \end{aligned}$$

Using the extension by zero in (18), we can write

35 $$\begin{aligned} {\varPi }^{\otimes 2}_{k}:=\sum _{\begin{array}{c} i+j=k\\ i,j=0, 1 \end{array}} {\varPi }_i\otimes {\varPi }_j, \end{aligned}$$

since for $u\in L^2{\varLambda }^{i'}$, $v\in L^2{\varLambda }^{j'}$, with $i'+j'=k$, we have

36 $$\begin{aligned} {\varPi }^{\otimes 2}_{k}(u\otimes v) = \sum _{\begin{array}{c} i+j=k\\ i,j=0,1 \end{array}} {\varPi }_{i}(u)\otimes {\varPi }_{j}(v) = {\varPi }_{i'}(u)\otimes {\varPi }_{j'}(v), \end{aligned}$$

Definition (36) extends by linearity and density to all elements of $(L^2{\varLambda }^{\otimes 2})^{k}$.

The definition of quasi-interpolation operators in the form (35) generalizes to the tensor product of any finite number of quasi-interpolation operators by the following construction. We start defining the domain of this tensor product operator in *n* dimensions:

$$\begin{aligned} (L^2{\varLambda }^{\otimes n})^{k} =\bigoplus _{{\mathbf {i}}\in \chi _k} L^2{\varLambda }^{i_1}\otimes \cdots \otimes L^2{\varLambda }^{i_n}, \quad k=0,\ldots ,n. \end{aligned}$$

#### Remark 6

Note that, by Fubini’s theorem, the following isomorphisms hold:

$$\begin{aligned} (L^2{\varLambda }^{\otimes n})^{0}({\mathscr {I}}_\varrho ^{\times n})&= \underbrace{L^2{\varLambda }^0({\mathscr {I}}_\varrho ) \otimes \cdots \otimes L^2{\varLambda }^0({\mathscr {I}}_\varrho )}_{n\ \text {times}}\simeq L^2{\varLambda }^0({\mathscr {I}}_\varrho ^{\times n}),\\ (L^2{\varLambda }^{\otimes n})^{n}({\mathscr {I}}_\varrho ^{\times n})&=\underbrace{L^2{\varLambda }^1({\mathscr {I}}_\varrho ) \otimes \cdots \otimes L^2{\varLambda }^1({\mathscr {I}}_\varrho )}_{n\ \text {times}}\simeq L^2{\varLambda }^n({\mathscr {I}}_\varrho ^{\times n}). \end{aligned}$$

#### Definition 4

Given the commuting quasi-interpolation operators in one dimension from Sect. 5.3, we define the tensor product quasi-interpolator in *n* dimensions, for $k=0,1,\ldots ,n$, as

37 $$\begin{aligned} {\varPi }^{\otimes n}_{k}:(L^2{\varLambda }^{\otimes n})^{k}\rightarrow ({\mathbb {P}}{\varLambda }^{\otimes n})^{k}, \qquad {\varPi }^{\otimes n}_{k}:= \sum _{{\mathbf {i}}\in \chi _k}{\varPi }_{i_1}\otimes \cdots \otimes {\varPi }_{i_n}. \end{aligned}$$

The operator ${\varPi }^{\otimes n}_{k}$ applies to the tensor product of rank-one functions as follows: given $u_1\otimes \cdots \otimes u_n\in (L^2{\varLambda }^{\otimes n})^{k}$, with $u_j\in L^2{\varLambda }^{i_j}$ and ${\mathbf {i}}=(i_1,\ldots ,i_n)\in \chi _k$, it holds

38 $$\begin{aligned} {\varPi }^{\otimes n}_{k}(u_1\otimes \cdots \otimes u_n) = \sum _{\begin{array}{c} {\mathbf {i}}'\in \chi _k \end{array}} {\varPi }_{i'_1}(u_1)\otimes \cdots \otimes {\varPi }_{i'_n}(u_n) ={\varPi }_{i_1}(u_1)\otimes \cdots \otimes {\varPi }_{i_n}(u_n),\nonumber \\ \end{aligned}$$

where in the second equality we have used (18). Definition (38) extends by linearity and density to all elements of $(L^2{\varLambda }^{\otimes n})^{k}$.

#### Lemma 7

*The quasi-interpolation operator* ${\varPi }^{\otimes n}_{k}$ *defined in* (37) *is bounded in* $L^2({\mathscr {I}}_\varrho ^{\times n})$.

#### Proof

Lemma 6 states that the tensor product of bounded operators on Hilbert spaces is bounded, with constant given as product of the individual constants. Then, it holds:

$$\begin{aligned} \left\| {\varPi }^{\otimes n}_{k}\right\| _{}&\le \sum _{{\mathbf {i}}\in \chi _k} \left\| {\varPi }_{i_1}\otimes \cdots \otimes {\varPi }_{i_n}\right\| _{} = \sum _{{\mathbf {i}}\in \chi _k} \left\| {\varPi }_{i_1}\right\| _{}\cdots \left\| {\varPi }_{i_n}\right\| _{}\\&\le \left( \sum _{{\mathbf {i}}\in \chi _k} 1\right) C_{\varPi }^n = \left( \begin{array}{c} n\\ k\end{array}\right) C_{\varPi }^n, \end{aligned}$$

where $C_{\varPi }=\max \{C_{{\varPi }_0},C_{{\varPi }_1}\}$, the constants $C_{{\varPi }_0}, C_{{\varPi }_1}$ being introduced in Theorem 1. $\square$

The following lemma shows that ${\varPi }^{\otimes n}_{k}$ is a co-chain operator.

#### Lemma 8

*The tensor product operator* ${\varPi }^{\otimes n}_{k}$ *commutes with the exterior derivative. More precisely, for* $u\in (H{\varLambda }^{\otimes n})^k$, *there holds*

$$\begin{aligned} {\varPi }^{\otimes n}_{k+1}(d^k u) = d^k{\varPi }^{\otimes n}_{k} u \end{aligned}$$

#### Proof

We start proving the result on rank-one functions $u_1\otimes \cdots \otimes u_k\in (L^2{\varLambda }^{\otimes n})^{k}$, with $u_j\in L^2{\varLambda }^{i_j}$, ${\mathbf {i}}=(i_1,\ldots ,i_n)\in \chi _k$. Using (31), the linearity of ${\varPi }^{\otimes n}_{k}$,  (18) and Lemma 5, there holds

$$\begin{aligned} {\varPi }^{\otimes n}_{k+1}(d^k(u_1\otimes \cdots \otimes u_n))&= {\varPi }^{\otimes n}_{k+1} \left( \sum _{j=1}^n\theta _j\, u_1\otimes \cdots \otimes d^{i_j}u_j\otimes \cdots \otimes u_n\right) \\&= \sum _{j=1}^n\theta _j {\varPi }^{\otimes n}_{k+1}(u_1\otimes \cdots \otimes d^{i_j}u_j\otimes \cdots \otimes u_n)\\&= \sum _{j=1}^n\theta _j {\varPi }_{i_1}(u_1)\otimes \cdots \otimes {\varPi }_{i_j+1}(d^{i_j}u_j)\otimes \cdots \otimes {\varPi }_{i_n}(u_n)\\&= \sum _{j=1}^n\theta _j {\varPi }_{i_1}(u_1)\otimes \cdots \otimes d^{i_j}{\varPi }_{i_j}(u_j)\otimes \cdots \otimes {\varPi }_{i_n}(u_n)\\&= d^k({\varPi }^{\otimes n}_{k}(u_1\otimes \cdots \otimes u_n)). \end{aligned}$$

The result extends by linearity and density to all elements of the tensor product space $(L^2{\varLambda }^{\otimes n})^{k}$. $\square$

#### Remark 7

A similar tensor product construction has been applied to bounded cochain projectors in [7].

#### Remark 8

We did not address the question of boundary conditions in our construction. This remark discusses how to define a quasi-interpolation operator which preserves boundary data and implications on regularity. We do not claim here that we have found a viable solution, but rather provide some discussion points for further research.

Formally, it is very simple to modify our tensor product weighted node functionals by removing the integral over the direction normal to the boundary and only averaging over points on the boundary itself, a construction similar to the operator in [28]. But this is only well-posed, if the trace of the interpolated function on the boundary exists. Clearly, such an interpolator cannot be bounded on $L^2({\varOmega })$. It is bounded on $H^1({\varOmega })$, if no derivatives are involved. Node functionals with tangential derivatives can be dealt with by integration by parts in the same way as in (19), such that for these no additional regularity is needed.

If the node functionals on the boundary involve normal derivatives the interpolation operator constructed in this way is not even bounded on $H^1({\varOmega })$. Thus, we are thrown back to the $L^2$-cochain complex. On the other hand, for boundary values of the normal derivative in $L^2(\partial {\varOmega })$, we can replace the integral over the interpolated function, which is typically the unknown solution, by the integral over the boundary data. This is well-posed, and it is consistent if the boundary value problem is well-posed. The functional analytic foundations of this technique for the case $k=n-1$ can be found in [16, Section 2]. A generalization to arbitrary *k* is still lacking.

## Higher-order polynomial spaces

In this section, we repeat the construction of Sects. 4 and 5 to obtain commuting quasi-interpolation operators for finite elements of arbitrary polynomial order.

### Canonical interpolation operators

The canonical commuting interpolation operators in Sect. 4 extend to higher order polynomial spaces in a straightforward way, if we introduce additional node functionals and corresponding basis functions. To this end, let $\ell _m \in {\mathbb {P}}_m$ be the Legendre polynomial of degree *m* on the interval ${\mathscr {I}}$, normalized such that $\ell _m(1) = 1$. Then, the sequence $\{\ell _m\}_{m=0,\dots }$ is mutually $L^2({\mathscr {I}})$-orthogonal. We also introduce the integrated and twice integrated Legendre polynomials

$$\begin{aligned} L_m(x) = \int _0^x \ell _m(t)\,dt, \qquad K_m(x) = \int _0^x L_m(t)\,dt. \end{aligned}$$

We recall the well known relation

39 $$\begin{aligned} 2(2m+1) L_m(x) = \ell _{m+1}(x) - \ell _{m-1}(x), \end{aligned}$$

which implies the following properties:

(i) $L_m(0) = L_m(1) = 0$ and equivalently $K^\prime _m(0) = K^\prime _m(1) = 0$ for $m\ge 1$, since $L_m$ is the difference of two Legendre polynomials of equal parity.
(ii) $K_m(0) = K_m(1) = 0$, for $m\ge 2$, since $K_m$ ($m\ge 2$) is the integral of a function with zero mean vanishing at the interval ends.

We define the node functionals for higher-order polynomial finite elements ${\mathbb {P}}_m{\varLambda }^0$ and ${\mathbb {P}}_{m-1}{\varLambda }^1$ by the following interpolation conditions.

- For $${\mathbb {P}}_m{\varLambda }^0({\mathscr {I}})$$, use
  40 $$\begin{aligned} \begin{aligned} {\mathscr {N}}^0_1 (u)&= u'(0)&\qquad {\mathscr {N}}^0_{m+1} (u)&= u(0) + u(1)\\ {\mathscr {N}}^0_2 (u)&= u'(1)\\ {\mathscr {N}}^0_{i+3}(u)&= \int _{{\mathscr {I}}} \ell _i u^\prime \,dx,&i&=0,\dots ,m-3. \end{aligned} \end{aligned}$$
  While ${\mathscr {N}}^0_1$ and ${\mathscr {N}}^0_2$ are identical to (10), we replaced ${\mathscr {N}}^0_3$ by an integral over $u'$, which evaluates to the same as the original. The functional ${\mathscr {N}}^0_4$ stayed the same, but now received the index $m+1$ to be conforming with Lemma 1. In what follows, we will refer to $\{{\mathscr {N}}^0_1,{\mathscr {N}}^0_2,{\mathscr {N}}^0_3,{\mathscr {N}}^0_{m+1}\}$ as the original node functionals and introduce the corresponding index set $J_o = \{1,2,3,m+1\}$.
- For ${\mathbb {P}}_{m-1}{\varLambda }^1({\mathscr {I}})$, introduce the node functionals
  41 $$\begin{aligned} \begin{aligned} {\mathscr {N}}^1_1 (v)&= v(0)\\ {\mathscr {N}}^1_2 (v)&= v(1) \end{aligned} \qquad {\mathscr {N}}^1_{i+3} (v) = \int _{{\mathscr {I}}} \ell _i v\,dx\qquad i=0,\dots ,m-3. \end{aligned}$$

Moreover, we choose the basis $\{\varphi ^0_i\}$ and $\{\varphi ^1_i\}$ for the spaces ${\mathbb {P}}_m={\mathbb {P}}_m{\varLambda }^0({\mathscr {I}})$ and ${\mathbb {P}}_{m-1}={\mathbb {P}}_{m-1}{\varLambda }^1({\mathscr {I}})$, respectively, as follows:

- $\varphi ^0_1,\ \varphi ^0_2,\ \varphi ^0_3$ and $\varphi ^0_{m+1}$ are chosen identical to (13). The remaining polynomials are chosen as
  42 $$\begin{aligned} \varphi ^0_i(x) = K_{i-2}(x),\qquad i=4,\dots ,m, \end{aligned}$$
  where we note that $K_{i-2}$ has degree *i*.
- $\varphi ^1_1,\ \varphi ^1_2$ and $\varphi ^1_3$ are chosen identical to (12). The remaining polynomials are chosen as
  43 $$\begin{aligned} \varphi ^1_i(x) = L_{i-2}(x),\qquad i=4,\dots ,m, \end{aligned}$$
  where we note that $L_{i-2}$ has degree $i-1$.

The canonical interpolation operators, as before $I_0:{\varLambda }^0({\mathscr {I}})\rightarrow {\mathbb {P}}_m{\varLambda }^0({\mathscr {I}})$ and $I_1:{\varLambda }^1({\mathscr {I}})\rightarrow {\mathbb {P}}_{m-1}{\varLambda }^1({\mathscr {I}})$ are then defined as

$$\begin{aligned} I_0 u(x) = \sum _{i=1}^{m+1} {\mathscr {N}}^0_i(u) \,\varphi ^0_i(x), \qquad I_1 v(x) = \sum _{j=1}^m {\mathscr {N}}^1_j (v) \,\varphi ^1_j(x). \end{aligned}$$

#### Lemma 9

*The space* ${\mathbb {P}}_m={\mathbb {P}}_m{\varLambda }^0({\mathscr {I}})$ *with the node functionals in* (40) *forms a unisolvent finite element.*

#### Proof

First, we note that the dimension of ${\mathbb {P}}_m$ equals the number of node functionals. Thus, it is sufficient to show that for $p\in {\mathbb {P}}_m$ there holds

44 $$\begin{aligned} \biggl [\quad {\mathscr {N}}^0_i(p) = 0\quad \forall \,i = 1,\dots ,m+1\quad \biggr ] \quad \Longrightarrow \quad \Bigl [\;p\equiv 0\;\Bigr ]. \end{aligned}$$

We show (44) by writing *p* as linear combination of the basis $\{\varphi ^0_i\}$:

$$\begin{aligned} p(x) = \sum _{i=1}^{m+1} \alpha _i \varphi ^0_i(x). \end{aligned}$$

Since $K_i$ for $i\ge 2$ has double roots at 0 and 1, there holds

$$\begin{aligned} {\mathscr {N}}^0_i(\varphi ^0_j) = 0 \qquad i\in J_o, \quad j=4,\dots ,m. \end{aligned}$$

Therefore, by standard Hermitian interpolation conditions, we obtain

$$\begin{aligned} \Bigl [\;{\mathscr {N}}^0_i(p) = 0 \quad \forall i\in J_o\;\Bigr ] \quad \Longrightarrow \quad \Bigl [\;\alpha _i = 0\quad \forall i\in J_o\;\Bigr ]. \end{aligned}$$

For the remaining coefficients we prove $\alpha _i = 0$ by induction. First note for $i=1,\dots ,m-3$

$$\begin{aligned} {\mathscr {N}}^0_{i+3}(p)&= \sum _{j=4}^{m} \int _{{\mathscr {I}}} \ell _i (x) \alpha _j K^\prime _{j-2}(x)\,dx= \sum _{j=4}^{m} \int _{{\mathscr {I}}} \ell _i (x) \alpha _j L_{j-2}(x)\,dx\\&{\mathop {=}\limits ^{(38)}} \sum _{j=4}^{m} \tfrac{\alpha _j}{2(2j-3)} \int _{{\mathscr {I}}} \ell _i (x) \bigl (\ell _{j-1}(x) - \ell _{j-3}(x)\bigr )\,dx. \end{aligned}$$

Thus, by orthogonality of the Legendre polynomials

$$\begin{aligned} {\mathscr {N}}^0_4(p) = \tfrac{\alpha _4}{10} \int _{{\mathscr {I}}} \ell _1^2 (x) \,dx. \end{aligned}$$

We conclude that ${\mathscr {N}}^0_4(p)=0$ implies $\alpha _4 = 0$. Assume now that $4<n<m$, and $\alpha _k=0$ for all $1 \le k \le n-1$. Then,

$$\begin{aligned} {\mathscr {N}}^0_{n}(p)&= \sum _{j=n}^{m} \alpha _j\tfrac{1}{2(2j-3)} \int _{{\mathscr {I}}} \ell _{n-3} (x) \bigl (\ell _{j-1}(x) - \ell _{j-3}(x)\bigr )\,dx\\&= \tfrac{\alpha _n}{2(2n-3)} \int _{{\mathscr {I}}} \ell _{n-3}^2(x) \,dx. \end{aligned}$$

Hence, $${\mathscr {N}}^0_n(p)=0$$ implies $$\alpha _n = 0$$. $$\square $$

By a similar, but simpler argument, we can prove

#### Lemma 10

*The space* ${\mathbb {P}}_{m-1}={\mathbb {P}}_{m-1}{\varLambda }^1({\mathscr {I}})$ *with the node functionals in* (41) *forms a unisolvent finite element.*

Analog to Lemma 3, we have the following lemma.

#### Lemma 11

*The following diagram commutes:*
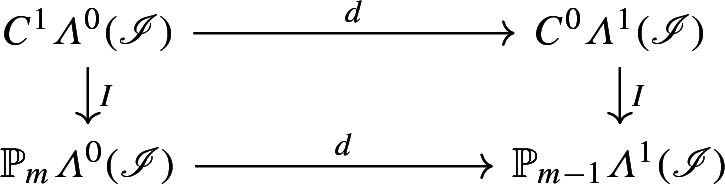
*that is, for every* $u\in C^1{\varLambda }^0({\mathscr {I}})$, *there holds*

$$\begin{aligned} d^0 I_0 u = I_1 d^0 u. \end{aligned}$$

#### Proof

We show that Lemma 1 applies to the node functionals (40) and (41), and the basis functions (42) and (43). Since the functionals ${\mathscr {N}}^0_i$ with $i\in J_o$ and ${\mathscr {N}}^1_i$ with $i\in \{1,2,3\}$ have not changed except for the reformulation of ${\mathscr {N}}^0_3$, the result of Lemma 3 still applies to those. For the remaining ones, we have

$$\begin{aligned} {\mathscr {N}}^1_{i+3}(du) = \int _{{\mathscr {I}}} \ell _i du\,dx= \int _{{\mathscr {I}}} \ell _i u'\,dx= {\mathscr {N}}^0_{i+3}(u), \qquad i=1,\dots ,m-3. \end{aligned}$$

We conclude observing that, by definition, $d\varphi ^0_i=\varphi ^1_i$ for $i=1,\ldots ,m$, and $d\varphi ^0_{m+1}=0$ holds. $\square$

### Quasi-interpolation operators

Again, we introduce node functionals on the perturbed interval ${{\tilde{{\mathscr {I}}}}}_{y_l,y_r}$. To this end, it is sufficient to define transformed versions of the new node functionals in (40) and (41), since the original ones with index in $J_o$ are transformed as before (see Eqs. (14) and (15)). To this end, let $\{{{\tilde{\ell }}}_i\}$ be the sequence of orthogonal polynomials on ${{\tilde{{\mathscr {I}}}}}_{y_l,y_r}$, normalized such that ${{\tilde{\ell }}}_i(y_r) = 1$. The transformed node functionals are:

$$\begin{aligned} \widetilde{{\mathscr {N}}^0_{i+3}}(u)&= \int _{{{\tilde{{\mathscr {I}}}}}_{y_l,y_r}} {{\tilde{\ell }}}_i({\tilde{x}}) u'({\tilde{x}})\,d{\tilde{x}},\\ \widetilde{{\mathscr {N}}^1_{i+3}}(u)&= \int _{{{\tilde{{\mathscr {I}}}}}_{y_l,y_r}} {{\tilde{\ell }}}_i({\tilde{x}}) u({\tilde{x}})\,d{\tilde{x}}. \end{aligned}$$

Note that by this definition, we still have the commutation property

$$\begin{aligned} \widetilde{{\mathscr {N}}^1_{i+3}}(d u) = \widetilde{{\mathscr {N}}^0_{i+3}}(u) \qquad i=0,\dots ,m-3. \end{aligned}$$

We can now define the weighted node functionals as in (16), and the quasi-interpolation operator as in (17). Lemma 1 applies, hence the quasi-interpolation operators commute with the exterior derivative. Moreover, the tensor product construction of Sect. 6 leads to commuting quasi-interpolation operators with values in $({\mathbb {P}}{\varLambda }^{\otimes n})^k$ as in (34), where ${\mathbb {P}}{\varLambda }^0={\mathbb {P}}_m{\varLambda }^0({\mathscr {I}})$ and ${\mathbb {P}}{\varLambda }^1={\mathbb {P}}_{m-1}{\varLambda }^1({\mathscr {I}})$.

## Conclusions

The one-dimensional $H^1$-conforming finite element cochain complex based on cubic and higher-order polynomials, and its quasi-interpolation operators were introduced. The tensor product construction was employed to derive (i) $H^1$-conforming finite element cochain complexes on meshes with Cartesian mesh cells of arbitrary dimension; (ii) $L^2$-stable quasi-interpolation cochain operators.

The construction principle in Sect. 8 can be generalized to higher differentiability by adding more derivative degrees of freedom at the interval edges and adjusting the remaining degrees of freedom. The argument using integration by parts in Theorem 1 remains valid if applied multiple times and yields $L^2$-stable quasi-interpolation operators also for this case.

When we refer to meshes with Cartesian mesh cells, we mean that all boundaries of mesh cells are axiparallel. While this is more general than a Cartesian mesh and allows for domains with nontrivial topology, it is nevertheless very restrictive. Lifting this condition is not trivial though. First, it is known that the relation $dV^k\subset V^{k+1}$ does not hold anymore directly for finite element spaces, but only after applying an additional Riesz isomorphism, see [3]. Second, the tensor product construction of degrees of freedom requires coordinate systems in vertices, which are consistent over all attached cells. This can be achieved at “regular vertices”, see [1], but it is not clear, whether a construction at irregular vertices can be obtained.

## Figures and Tables

**Fig. 1:**
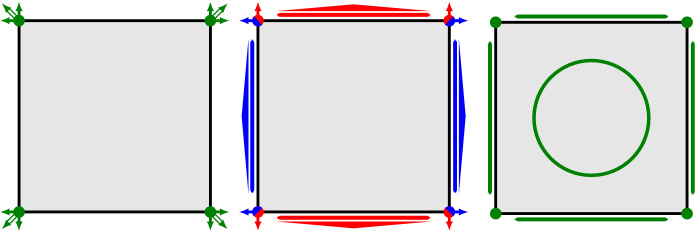
Node functionals of the vector proxies in two dimensions for $k=0,1,2$ from left to right. Bullets are point values, arrows are derivatives in a point and double diagonal arrows are mixed second derivatives. Lines and the circle are integrals in one and two dimension, respectively. Flat triangles are integrals over the normal derivative. In the center, blue and red degrees of freedom refer to the horizontal and vertical vector components, respectively

## Appendix: Vector proxies

Based on the identifications of the spaces $\mathrm{Alt}^0\,{\mathbb {R}}^n$ and ${\text {Alt}}^{n}{\mathbb {R}}^n$ with ${\mathbb {R}}$, as well as of ${\text {Alt}}^1{\mathbb {R}}^n$ and ${\text {Alt}}^{n-1}{\mathbb {R}}^n$ with ${\mathbb {R}}^n$, natural correspondences may be established between spaces of differential 0 and *n* forms with scalar-valued fields, as well as spaces of differential 1 and $(n-1)$ forms with vector-valued fields. The associated fields are known as proxy fields. In the following, we detail the tensor product FE space of differential forms (34) for $n=2$ and $k=0,1,2$ and the vector proxy complex

$$\begin{aligned} 0 \xrightarrow {\;\;\subset \;\;}{\mathbb {R}}\xrightarrow {\;\;\subset \;\;} H^1({\mathscr {I}}\times {\mathscr {I}}) \xrightarrow {\;\;\nabla \times \;\;} H^{\text {div}}({\mathscr {I}}\times {\mathscr {I}}) \xrightarrow {\; \nabla \cdot \;} L^2({\mathscr {I}}\times {\mathscr {I}}) \xrightarrow {\;\;\;\;} 0. \end{aligned}$$

The commuting degrees of freedom in Eq.  (10) are only used for analysis and complicate the discussion below considerably. Therefore, we employ Remark 2 to replace ${\mathscr {N}}_3^0$ and ${\mathscr {N}}_4^0$ by the values in the end points. We will abuse the notation below and use the same symbol for these simplified degrees of freedom.

### Case $k=0$.

The space $({\mathbb {P}}{\varLambda }^{\otimes 2})^{0}({\mathscr {I}}\times {\mathscr {I}})={\mathbb {P}}_3{\varLambda }^0({\mathscr {I}})\otimes {\mathbb {P}}_3{\varLambda }^0({\mathscr {I}})$ is identified with the scalar proxy field ${\mathbb {P}}_3({\mathscr {I}})\otimes {\mathbb {P}}_3({\mathscr {I}})$, also denoted by ${\mathbb {P}}_{3,3}({\mathscr {I}}\times {\mathscr {I}})$. It has cardinality 16. The node functionals for ${\mathbb {P}}_{3,3}({\mathscr {I}}\times {\mathscr {I}})$ are the tensor product of node functionals for ${\mathbb {P}}_3({\mathscr {I}})$. For example, the four node functionals $\{{\mathscr {N}}^0_1\otimes {\mathscr {N}}^0_h\}_{h=1,\ldots ,4}$ are defined on $u(x,y)\in {\mathbb {P}}_{3,3}({\mathscr {I}}\times {\mathscr {I}})$ as

$$\begin{aligned} \begin{aligned} ({\mathscr {N}}^0_1\otimes {\mathscr {N}}^0_1) (u)&= \frac{\partial ^2 u}{\partial x\partial y }(0,0) \qquad&({\mathscr {N}}^0_1\otimes {\mathscr {N}}^0_3) (u)&= \frac{\partial u}{\partial x}(0,0)\\ ({\mathscr {N}}^0_1\otimes {\mathscr {N}}^0_2) (u)&= \frac{\partial ^2 u}{\partial x\partial y }(0,1)&({\mathscr {N}}^0_1\otimes {\mathscr {N}}^0_4) (u)&= \frac{\partial u}{\partial x}(0,1). \end{aligned} \end{aligned}$$

Altogether, the node functionals for ${\mathbb {P}}_{3,3}({\mathscr {I}}\times {\mathscr {I}})$ are (see also Fig. 1):

1. The mixed second derivatives in each vertex
2. The two components of the gradient in each vertex
3. The function values in each vertex

In summary, we obtain the finite element of Bogner, Fox, and Schmit [6].

### Case $k=1$.

The space $({\mathbb {P}}{\varLambda }^{\otimes 2})^{1}({\mathscr {I}}\times {\mathscr {I}})=({\mathbb {P}}_3{\varLambda }^0({\mathscr {I}})\otimes {\mathbb {P}}_2{\varLambda }^1({\mathscr {I}}))\oplus ({\mathbb {P}}_2{\varLambda }^1({\mathscr {I}})\otimes {\mathbb {P}}_3{\varLambda }^0({\mathscr {I}}))$ is identified with the proxy vector field $({\mathbb {P}}_3({\mathscr {I}})\otimes {\mathbb {P}}_2({\mathscr {I}}))\oplus ({\mathbb {P}}_2({\mathscr {I}})\otimes {\mathbb {P}}_3({\mathscr {I}}))$, also denoted by ${\mathbb {P}}_{3,2}({\mathscr {I}}\times {\mathscr {I}})\oplus {\mathbb {P}}_{2,3}({\mathscr {I}}\times {\mathscr {I}})$. This is the tensor product Raviart-Thomas space of corresponding order on the square. Both spaces ${\mathbb {P}}_{3,2}({\mathscr {I}}\times {\mathscr {I}})$ and ${\mathbb {P}}_{2,3}({\mathscr {I}}\times {\mathscr {I}})$ have cardinality 12.

Again, the node functionals are constructed as tensor products. For example, the three nodal functionals $\{{\mathscr {N}}^0_1\otimes {\mathscr {N}}^1_h\}_{h=1,2,3}$ are defined on $v(x,y)\in {\mathbb {P}}_{3,2}({\mathscr {I}}\times {\mathscr {I}})$ as

$$\begin{aligned} \begin{aligned} ({\mathscr {N}}^0_1\otimes {\mathscr {N}}^1_1)(v)&= \frac{\partial v}{\partial x}(0,0)\\ ({\mathscr {N}}^0_1\otimes {\mathscr {N}}^1_2)(v)&= \frac{\partial v}{\partial x}(0,1) \end{aligned} \qquad ({\mathscr {N}}^0_1\otimes {\mathscr {N}}^1_3)(v) = \int _0^1 \frac{\partial v}{\partial x}(0,y)\, dy. \end{aligned}$$

For each of the four edges, we obtain the following set of six node functionals (see also Fig. 1):

1. The values of the normal derivative of the normal component in the two end points of the edge
2. The integral of the normal derivative of the normal component over the edge
3. The function values of the normal component in the two end points of the edge
4. The integral of the normal component over the edge

This is the element of Austin, Manteuffel, and McCormick [5] with the degrees of freedom of Kanschat and Sharma [19].

### Case $k=2$.

The space $({\mathbb {P}}{\varLambda }^{\otimes 2})^{2}({\mathscr {I}}\times {\mathscr {I}})={\mathbb {P}}_2{\varLambda }^0({\mathscr {I}})\otimes {\mathbb {P}}_2{\varLambda }^0({\mathscr {I}})$ is identified with the scalar proxy field ${\mathbb {P}}_2({\mathscr {I}})\otimes {\mathbb {P}}_2({\mathscr {I}})$, also denoted by ${\mathbb {P}}_{2,2}({\mathscr {I}}\times {\mathscr {I}})$. It has cardinality 9. Node functionals for ${\mathbb {P}}_{2,2}({\mathscr {I}}\times {\mathscr {I}})$ are ${\mathscr {N}}^1_\ell \otimes {\mathscr {N}}^1_h$, for $\ell , h=1,2,3$. For example,

$$\begin{aligned} \begin{aligned} ({\mathscr {N}}^1_1\otimes {\mathscr {N}}^1_1)(w)&= w(0,0), \quad ({\mathscr {N}}^1_1\otimes {\mathscr {N}}^1_3)(w) = \int _0^1 w(0,y)\,dy,\\ ({\mathscr {N}}^1_1\otimes {\mathscr {N}}^1_2)(w)&= w(0,1),\quad ({\mathscr {N}}^1_3\otimes {\mathscr {N}}^1_3)(w) = \iint _0^1 w(x,y)\, dx\, dy, \end{aligned} \end{aligned}$$

for $w(x,y)\in {\mathbb {P}}_{2,2}({\mathscr {I}}\times {\mathscr {I}})$. Altogether, the node functionals for ${\mathbb {P}}_{2,2}({\mathscr {I}}\times {\mathscr {I}})$ are (see also Fig. 1):

1. The function values at the four vertices
2. The integrals of function values over each edge
3. The integral of the function over the square

This is the standard continuous ${\mathbb {Q}}_2$ element with moment degrees of freedom.

## Comparison to other elements

The construction of the isogeometric methods in [8] and [9] is done by tensor products of one-dimensional elements. Thus, for the same polynomial degree and the same continuity, they have the same number of degrees of freedom on a single patch.

In [21] two finite element pairs for velocity and pressure have been proposed. For the first, the discrete velocity lives in the space ${\mathbb {P}}_{3,2}({\mathscr {I}}\times {\mathscr {I}})\oplus {\mathbb {P}}_{2,3}({\mathscr {I}}\times {\mathscr {I}})$, where ${\mathbb {P}}_{3,2}({\mathscr {I}}\times {\mathscr {I}})$, the same Raviart-Thomas space as above for $k=1$. There, this space is equipped with the following set of degrees of freedom:

1. On each edge, the value of the function and the normal derivative of the normal component at the end points
2. On each edge, the function evaluation in the center point
3. Function evaluation at two interior points for each velocity component

The pressure space ${\mathbb {P}}_{2,2}({\mathscr {I}}\times {\mathscr {I}})$ is equipped with the following set of node functionals

1. The function evaluation in each vertex
2. Five internal degrees of freedom

The second pair in [21] is a reduced version with smaller function spaces. We compare the number of degrees of freedom involved for the pure tensor product elements and the Neilan/Sap pairs in Table 1. Nevertheless, the value of this comparison is limited, since it must be put in relation with the approximation properties and ease of implementation.

**Table 1:** Comparison of the number of degrees of freedom per cell for the tensor product elements in this article and the two elements by Neilan and Sap. Comparison for a single cell and for an infinite lattice in two dimensions

Element	Single cell		Asymptotically	
	Velocity	Pressure	Velocity	Pressure
Tensor product	24	9	8	4
Neilan/Sap I	24	9	10	6
Neilan/Sap II	20	5	6	2
